# Understanding the Needs of Young People Who Engage in Self-Harm: A Qualitative Investigation

**DOI:** 10.3389/fpsyg.2019.02916

**Published:** 2020-01-10

**Authors:** Sarah E. Hetrick, Aruni Subasinghe, Kate Anglin, Laura Hart, Amy Morgan, Jo Robinson

**Affiliations:** ^1^Department of Psychological Medicine, The University of Auckland, Auckland, New Zealand; ^2^Centre for Youth Mental Health, University of Melbourne, Melbourne, VIC, Australia; ^3^Orygen, The National Centre of Excellence in Youth Mental Health, University of Melbourne, Melbourne, VIC, Australia; ^4^School of Psychology and Public Health, College of Science, Health and Engineering, La Trobe University, Melbourne, VIC, Australia; ^5^Centre for Mental Health, Melbourne School of Population and Global Health, University of Melbourne, Melbourne, VIC, Australia

**Keywords:** self-harm, suicide risk, young people, qualitative, interventions

## Abstract

Self-harm is common and associated with adverse outcomes. Research about the risk factors for self-harm has informed the field with regard to clinical interventions that should be delivered for young people who engage in self-harm. Missing is an in-depth understanding of what the triggers of an urge to self-harm might be, including in young people being treated with a clinical intervention. Therefore, there is little knowledge about what techniques young people find helpful to deal with urges to self-harm when they occur. This qualitative study engaged seven young people with lived experience of self-harm in semi-structured interviews about the immediate triggers of the urge to self-harm, and helpful strategies to manage this urge. Thematic analysis using a general inductive approach revealed distressing emotions and a sense of isolation as key themes, with other triggers associated with their induction. Highlighted was the wide range of situations and emotions that can be triggering, such that a further key theme was the idiosyncratic nature of the self-help strategies young people found helpful. Interventions that are developed to support young people who self-harm must address this complexity and findings highlight the need for young people to maintain some autonomy and control while being supported to connect with others for support. This research adds to the literature on self-help strategies to support young people in moments when they are experiencing distressing emotions, feel isolated, and have an urge to self-harm providing important insight to the prevention and intervention for self-harm among young people.

## Introduction

There is no standard definition of self-harm. For the purposes of this paper it is defined as intentional self-poisoning or self-injury irrespective of intent (Hawton et al., 2003). While variously referred to as non-suicidal self-injury (NSSI) and deliberate self-harm; the former has been criticized because the term assumes the absence of intent, but it is accepted that intent can be difficult to determine and those who engage in NSSI have higher rates of suicidal ideation (intent) and suicide attempt (Whitlock et al., 2006; Joiner et al., 2012), and the second due to self-harm often occurring in a dissociative state so that the person may not be completely aware of what they are doing (Robinson, 2017).

Self-harm is a critical issue affecting up to 25% of young people and can result in adverse outcomes including repetition of self-harm, suicide and mortality, mental health morbidity, poorer education and employment outcomes, and overall decreased quality of life, as well as being costly to treat (Fergusson et al., 2005; Harrington et al., 2006; Madge et al., 2008; Bergen et al., 2012; Clark et al., 2013; Mars et al., 2014; Robinson, 2016). The Second Australian Child and Adolescent Survey of Mental Health and Wellbeing reports that approximately 8% of young people aged between 12 and 17 years old engaged in self-harm without suicide intent in the previous 12 months. Rates were higher in those who identified as having any mental disorder (Lawrence et al., 2015; Zubrick et al., 2016). A New Zealand study examining rates of non-suicidal self-harm found life time rates of 48.7% (Garisch and Wilson, 2015). Compared to young people who don’t engage in self-harm, those who do are more likely to report suicidal ideation and plans for suicide and to report greater levels of emotional distress, difficulties with anger and low self-esteem, as well as antisocial behavior and health risk behaviors such as illicit drug use (Laye-Gindhu and Schonert-Reichl, 2005). Indeed, the rates of suicide attempt are higher in those who have engaged in NSSI (self-harm without suicide attempt) (Asarnow et al., 2011; Wilkinson et al., 2011; Guan et al., 2012; Klonsky et al., 2013; Knorr et al., 2016; Muehlenkamp et al., 2018). Self-harm in young people has been shown to have an impact on family relationships, wellbeing, and mental health (Morgan et al., 2013; Mars et al., 2014; Beckman et al., 2016).

There is a significant literature describing the risk factors for self-harm, an understanding of which is important for developing interventions. The literature about risk factors in young people has been summarized in a recent review highlighting previous abuse, mood disorder, borderline and other personality disorders, severity of symptoms of depression and hopelessness, and suicidal ideation (Witt et al., 2018) as key risks. Community-based studies similarly highlight mood disorder and history of abuse, and add bullying as more distal to engaging in self-harm, substance use, as well as psychological characteristics (Plener et al., 2015). Many interventions currently recommended for young people whose self-harm are clinic based, delivered by mental health clinicians, and primarily aimed to address the underlying mood and personality disorders (Hawton et al., 2015; Ougrin et al., 2015; Carter et al., 2016). These interventions are critical to deliver; however, many young people do not engage in traditional face-to-face mental health services (Whitlock et al., 2006; Ystgaard et al., 2009; Michelmore and Hindley, 2012; Doyle et al., 2015), and for those who do, the urge to self-harm can continue to occur, often and outside of the clinic setting, for some time. While these interventions include strategies specific to aiding a young person deal with the “in the moment” urge to self-harm, there is little literature exploring how young people perceive these strategies, and more particularly the extent to which the specific strategies designed to help them deal with the urge to self-harm are helpful or not.

To ensure young people have access to effective strategies when they experience the urge to self-harm, there is a need to move from describing generic risk factors to examining the triggers of this urge to self-harm. Understanding the phenomenology of self-harm from the perspective of young people with lived experience is critical (Brown and Kimball, 2013). In-depth insight into the perspectives of young people about the triggers of self-harm, and what might mitigate the urge to self-harm in the moment it occurs, provides opportunities for more powerful strategies, including those that young people can use themselves outside the clinic setting.

Therefore, the aims of this study were to identify both specific triggers of the urge to self-harm, and helpful strategies to manage this urge to engage in self-harm behaviors from the lived experience of young people.

## Materials and Methods

This study was given ethics approval by Melbourne Health Human Research Ethics Committee (HREC/15/MH/340).

Qualitative methods were used because these allow for an in-depth exploration of the lived experience of participants (Fossey et al., 2002). The raw data supporting the conclusions of this manuscript will be made available by the authors, without undue reservation, to any qualified researcher.

### Setting and Participants

The study was undertaken in a tertiary youth mental health service, Orygen Youth Health, and several secondary mental health services, headspace Glenroy and headspace Craigieburn (McGorry et al., 2014; Rickwood et al., 2014). These services are all located in Melbourne, Australia. Young people were recruited from the Youth Mood Clinic (Rice et al., 2017) at Orygen Youth Health and from the youth participation groups operating within Orygen Youth Health and the headspace services, and via chain-referral sampling where participants informed young people they thought might be eligible for inclusion. Young people were eligible if aged 18–25 years and had lived experience of suicidal ideation and/or self-harm (i.e., had experienced suicidal ideation or engaged in self-harm at some stage in their lives); but were ineligible if they had engaged in self-harm or had experienced severe suicidal ideation (frequent thoughts indicating an intent to kill themselves) in the past 3 months.

Recruitment was purposive and continued until saturation (no new data/concepts emerged) was reached. Guest et al. (2006) suggested that meta-themes often emerge within six interviews; we recruited seven young people.

### Procedure

Young people who were interested in the study were directed by their clinicians and the coordinators of the youth advisory groups to a webpage where they could register their interest. One of the investigators (SH), a clinical psychologist, contacted interested young people to describe the study, screen against the exclusion criteria, and to organize a time and place for written informed consent to be gained and the interview to be conducted. We reimbursed all participants for their time (AU $30 per hour).

We conducted semi-structured interviews, lasting between 40 and 60 min, using open-ended questions, that allowed flexibility and the opportunity to clarify and explore responses (Knox and Burkard, 2009). Initially, we provided a brief summary of the study, reiterating the aims, and affirmed participants as experts in their own experience to avoid the tendency of participants to omit information if they perceive that the researchers are the experts (Leech, 2002). The least potentially threatening questions were asked first and questions were worded in a non-threatening and non-judgmental manner, e.g., asked about use of alcohol or drugs rather than substance abuse (Leech, 2002). Informal prompts and restating (without reinterpreting) participants response allowed us to show interest in responses, confirm understanding, and encourage disclosure (Leech, 2002).

The interview schedule (see Online Supplementary Material) was developed on the basis of a systematic synthesis of both academic peer reviewed and gray online literature. Four electronic databases (PsycINFO, MEDLINE, EMBASE, and SCOPUS) and the United States, Australian, New Zealand, and United Kingdom Google platforms (google.com, google.com.au, google.co.nz, and google.co.uk) were searched. Within the academic literature controlled vocabulary (MeSH) terms were used in conjunction with keywords. The online search terms were developed iteratively until an acceptable level of relevance was found. Independent raters screened the retrieved studies and included English language studies relevant to participants aged 15–25 years, that were relevant to self-harm. There are differences in the terminology used to describe self-harm, particularly in regards to its underlying intent (Madge et al., 2008; Muehlenkamp et al., 2012); therefore, a range of terms were used in the search.

If webpages did not specify an age range for their intended audience, they were included; however, media sources such as video and audio were excluded. We extracted relevant data from the included studies and webpages into a structured template.

We developed a template for recording interview responses and included the questions and planned prompts that formed the semi-structured interview schedule, along with space to record each idea arising for each question.

### Analysis

We undertook a thematic analysis using a general inductive approach (Thomas, 2006), which allowed themes to be derived in the context of specific objectives. Raw data were condensed in the context of being guided by the objectives; however, findings were based on the raw data rather than *a priori* expectations derived from the objectives. The raw data were the responses of participants recorded by a researcher who sat in on each interview in our *a priori* designed template by a researcher.

Three principals of trustworthiness in qualitative research underpinned the analysis to ensure it was robust (Guba and Lincoln, 1985, 1989; Koch, 2006). The first principal was *credibility*, whereby participants were provided a summary of their responses at the completion of the interview to check for accuracy and to allow for further responding. Data were then independently coded by two researchers and discrepancies discussed and resolved by consensus. Interviews were audio recorded to ensure accuracy of direct quotes to exemplify themes. The second principal was *dependability*, whereby we followed an established procedure for analysis. This was the method of Braun and Clarke (2006), which includes six phases of analysis: (1) Familiarization with data including transcribing, reading, and re-reading transcripts; (2) Generating initial codes (on the basis of aims rather than research questions); (3) Searching for themes by collating codes into potential themes; (4) Reviewing themes and generating a thematic map of the analysis; (5) Defining and naming themes; and (6) Producing the report. *Transferability* or the degree to which the responses were representative was enhanced by recruiting from different services and we paid attention to the background and characteristics of recruited participants to ensure a range of experiences were included. Participants’ background and the recruitment and data collection processes used were consistently considered for their impact on results.

## Results

Saturation was achieved by the sixth interview, as expected according to the established procedure of Braun and Clarke (2006). In total, seven young people aged 18–24 years were interviewed (mean age 20.6, SD = 2.23); five females and two males. Three participants were past clients; two of headspace (Craigieburn; Glenroy) and one of Orygen Youth Health. Two participants were current clients, both of Orygen Youth Health and two participants had never been clients of either service but were receiving treatment from a private psychologist in one case and via a general practitioner in the other. Tables 1 and 2 provide a summary of the themes and subthemes that arose from the interviews.

**TABLE 1:** Themes and subthemes about triggers that arose from interviews.

**(1) Distressing emotions**
Helpless and guilty
Helpless and responsible for others’ self-harm
Guilty and unworthy of being depressed
Feeling like a burden on others
Felt unwanted and like a burden because parents had an unplanned pregnancy
Financial burden
Shame
Ashamed and embarrassed after alcohol use
Being overwhelmed
Lack of control
Confused because of mental health and emotions
Anxious because of school stressors
Anger
Angry because not “normal”
Rage because of bottled up emotions
Rage because there was no way to express emotions
**(2) Sense of isolation**
Family conflict
No friends at school
Did not belong
Stigma because of sexual orientation
People were not accepting of who they were
Mental illness
No support
**(3) Exposure to self-harm**
Contagion
Relationship with someone else who also engages in self-harm
Friends and peers who self-harm/attempt suicide
Visual triggers
Graphic images on Tumblr and Instagram
Normalized, reinforced, and encouraged behavior
Instagram quotes
Scars
Self-harm personal stories
Details of methods used
Empathizing with other’ negative experiences
**(4) Relationship difficulties**
Family
Unwanted and like a burden because parents had unplanned pregnancy
High parental expectations/disappointing parents
Lack of understanding
Lack of support
Lack of communication
**(4) Relationship difficulties *continued***
Friends and peers
Explaining self-harm scars
Bullying (including cyber-bullying)
Partners
Relationship with someone who also engages in self-harm
Lack of communication
Relationship break-up
**(5) Social comparison**
Graphic images on Tumblr
Identifying with others’ personal stories
Peers on social media
Perceived social norms
Academic performance to peers
**(6) School/work difficulties**
Exams
Deadlines
Year 12 pressure
Academic performance to peers
Hostile work environment
Rude customers and co-workers
Forced to attend work

**TABLE 2:** Themes and subthemes about self-help strategies that arose from interviews.

**(1) Idiosyncratic nature of self help**
Dependent on level of distress/mood
Some things don’t work on a bad day
Dependent on type of mental illness; symptoms
Dependent on personal interests
Depends on individual triggers
Depends on what particular activities you enjoy
Dependent on what point in time the strategy was used
Difficult if it is the first time you use it
Not useful early in course of illness/treatment
Dependent on environment/setting
In public, e.g., at school
At home, in bedroom
Dependent on cause of distress
Feeling worthless
Feeling lonely
Conflict with family/friends
**(2) The importance of distraction**
Distraction is idiosyncratic to individuals
Enjoyable/soothing activities
Immersive activities to divert very strong emotions
Activities to help release bottled up emotions
Matching self-help to type and strength of emotion
Help box with a range of options a young person knows helps them (as a reminder; and different things for different times)
Easy to forget things in the moment so need it all in one place
Sadness box/comfort box with personal things to make you feel good
Distraction from worry that underlies distress
Do something so you can put away the worry and come back to it later
**(3) Connectedness**
Overcome feelings of isolation
Overcoming sense of depersonalization
Feel validated/feel like others are also experiencing similar things
Gain another perspective
Assistance with self-soothing
Needed someone just to be there; not necessarily to do anything
Sense of safety in the presence of someone else
Able to acknowledge the issue
Someone else knows
Limits rumination
Various modes of connection
Online or phone help meant control over who gets your information
Face-to-face better for real connection
Barriers and challenges
Forums and chat lines can normalize self-harm
Fear of being a burden and impacting on others’ well-being
Not knowing how to describe feelings/thoughts
Difficulties initiating discussion
**(4) Change in the environment**
Being in public is safer
Consequences of being seen to self-harm in public
Structure and things to do
Removing self from trigger
Getting away from stressful situations
Getting away to be able to work through emotion and think about problem
Going and doing an activity like exercise
Improved mood
Distraction from negative thinking
Focusing on something else
Social connection
**(5) Mimicking strategies (e.g., snapping elastic band)**
Unhelpful in private but useful in public
Subtle for when in public
Doesn’t have same effect
Doesn’t give same feeling
Need real pain
Had to keep doing it
Didn’t stop the self-harm
Condescending and invalidating
Don’t acknowledge the distress
Need to work on cause of self-harm

### Precipitating Factors and Triggers for Self-Harm

From 48 different triggers identified by participants, six themes arose: (1) distressing emotions, (2) sense of isolation, (3) exposure to self-harm, (4) relationship difficulties, (5) social comparison, and (6) school/work difficulties.

The theme of distressing emotions was described by all participants and appeared to be considered the primary trigger for urges to self-harm, along with a sense of isolation, with other themes becoming triggers by causing distressing emotions. Participants noted that in some instances they felt responsible and helpless in response to others’ self-harm, which would trigger their own urges to self-harm: “*I felt responsible especially because I was really close to some of them, so it was knowing about them going through [tough times] I blamed myself*” (Pt 5). One participant felt guilty because she did not feel like she was worthy of being depressed because she had a good family and was not experiencing any major difficulties compared to others: “*These people have gone through actual stuff and I’m just a sad teenager*” (Pt 1). Participants described feeling like a burden: “*They [my parents] didn’t intend on having me so when they had to deal with me it was a huge financial stress*… *I would feel like it’s my fault I shouldn’t have been born*” (Pt 4). Feelings of shame and embarrassment after alcohol use were also identified as triggers; participants stated that self-harm was not triggered by being intoxicated but by emotions experienced following excessive alcohol use: “*[I] felt so embarrassed because I let her down*” (Pt 1). Participants described feeling overwhelmed because of their mental health and emotions: “*I was so confused*… *I didn’t know how to feel, and was so used to feeling worthless*” (Pt 7) and with school stress: “*So much pressure put on you, particularly in year 12. [there’s so much] pressure to do well*” (Pt 6). Frustration and anger were also described as triggers; related to theme 5 (social comparison) one participant described being triggered when she saw “normal” people on Facebook: “*I feel*… *angry because I see what they are doing that I can’t do*”. Related to theme 2 (sense of isolation), participants described how the inability to express emotions or concerns triggered feelings of anger and a sense of isolation: “*I would have rages at any time of the day*… *it was all the bottled up feelings that resulted in rages*” (Pt 5).

A sense of isolation (theme 2) was reported to arise from issues with friends or family and this triggered the urge to self-harm: “*They [my family] don’t love me anymore, I don’t mean anything to them*” (Pt 4); “*100% felt like I didn’t have support [from family]*… *I couldn’t find someone to confide in*” (Pt 6). Experiencing stigma as a result of a different sexual orientation and due to a mental illness was also reported to lead to a sense of isolation: “*Trying to maintain [a] relationship when people would tell me it’s disgusting and it’s wrong. It would be stressful. [they were] not accepting of who I am*” (Pt 4); “*Feeling isolated and alone probably comes from having bipolar because I feel like I’m alone in it and that no one can support me through it*” (Pt 3).

Exposure to self-harm (theme 3) included hearing about others’ experiences, seeing scars and images of self-harm, and reading personal self-harm stories; this was in the context of personal relationships: “*Having her [partner] around and seeing her do it, I felt the need to also do it*” (Pt 6) and more generally: “*I know a few people in year nine and year 10*… *hearing about it and knowing that others were doing it too was triggering*” (Pt 3). Visual content on online platforms like Tumblr and Instagram were noted as triggering: “*When I was sad sometimes I wouldn’t want to hurt myself because I was scared, so I would*… *look at those blogs, it felt normal and then I would do it [self-harm]*” (Pt 1). Seeing scars from self-harm, either on themselves or others, was also reported as triggering: “*When the cut would heal and it would start to scar, I would think ‘it’s going away, let’s do it again*”’ (Pt 1), and the scars of others “*Seeing other people’s open wounds and new scars [was triggering]*” (Pt 2). Particularly in the online environment, the personal stories of other people who engaged in self-harm could be triggering, particularly those with details about methods: “*That’s [method details] probably the worst thing*… *what you use should never be discussed*” (Pt 1).

Relationship difficulties (theme 4) among family, friends, or intimate partners were described as potentially triggering. Failing to meet parental expectations, and a young person’s perception that their family was unsupportive and unwilling to talk openly about problems was considered triggering: “*I was triggered a lot by my parents not wanting me to see someone about it [mental illness/self-harm]*” (Pt 3); “*They saw that I had self-harmed and they made fun of me more than supported me and that was really difficult*… *it was the feeling of worthlessness that came from my parents putting me down*” (Pt 4). Cyberbullying was also identified as a trigger: “*It was a continuation from high school, because there was no other way to get to me except Facebook, so bullying continued through that*” (Pt 5). Both unsupportive intimate relationships and breaking up with a partner were described as triggering: “*I had a partner who was very closed off and didn’t want to talk about his or my emotions*” (Pt 3); “*My first serious relationship*… *that was a trigger for me, when it all ended*… *I’ve never been a strong relationship person and the stress of it was getting to me*” (Pt 5).

The theme of social comparison included comparison of self-harm injuries: “*When I saw pictures online it’s [my cuts are] too small, it’s not like that [the images]*” (Pt 1) and of the self compared to perceived social norms “*Meeting those social criteria. you see things and you know you don’t fit into that social norm and feel bad about yourself*” (Pt 2). This in turn increased a sense of isolation (theme 2).

School and work difficulties (theme 6) were often spoken about as triggering, especially pressure at school, as already described, but work pressures were also described as triggering: “*The chefs were not kind to me at all*… *they would call me awful names and swear at me all the time*” (Pt 3).

### Helpful Strategies When Experiencing an Urge to Self-Harm

Five themes arose with regard to helpful strategies that could be used by young people when resisting the urge to self-harm: (1) idiosyncratic nature of self-help, (2) the importance of distraction, (3) connectedness, (4) change in the environment, and (5) mimicking strategies. Table 2 shows how individual triggers were collated into themes.

The primary theme to emerge was the idiosyncratic nature of self-help strategies that participants used. Participants stated that how helpful a strategy was depended on a range of factors, for example, mood, level of distress, personal interests, whether they were practiced at the strategy or not, and the setting (home alone, school): “*Different things work for different people and at different times/days and in different situations*…*depends on how bad your day is and what the situation is*” (Pt 2), “*First time I hated it but tried it when having a better day*… *and it calms me. So learning about it*… *I read about it*” (Pt 5). Participants noted that it was likely that how helpful a strategy was depended on the nature of the trigger and that it was important to have an understanding of your own triggers: “*About finding out what your triggers are and knowing these and setting boundaries for yourself*” (Pt 5). The remainder of the themes described specific strategies, and within each of these, participants continued to reflect on how their efficacy varied according to context, triggers, and other factors.

A range of distraction techniques (theme 2) were described, and the importance of having a diverse set of strategies, which could be drawn upon at different times, was noted: “*Need a tool box of resources to draw on when you need it at different times*… *A comfort box*… *to put personal things in that make you feel good*” (Pt 1); the importance of matching to the particular emotion or situation was highlighted: “*mine is mostly a build-up of energy, so I would go for a sprint down the street*… *If I had bottled up emotions and just couldn’t get them out*… *I would watch a sad movie and that would help me to cry*” (Pt 6).

Participants described the importance of connecting with others (theme 3) as a self-help strategy, and highlighted the types and purposes of connection. This included overcoming feelings of isolation: “*For me it’s about feeling real*… *and like I am not by myself*” (Pt 3), dealing with negative thoughts: “*we would talk about it for a little while and try to rationalize it*” (Pt 4), and increasing a sense of safety and being cared for: “*So if you can sit with someone*… *not even really talk to them*… *but just sit and be safe*.”(Pt 3). Participants also spoke about how they resisted connection: “*I have spoken to friends who then told my mother, which was the right thing, but at the time I was so angry and it made it worse*” (Pt 1), how hard engaging with others could be: “*Should have a pre written text that you can just automatically have sent out. Some of the barriers is not knowing what to say and being worried about how they will react*… *it’s hard to express myself*” (Pt 6). Participants highlighted the value of helplines and online forums because they could connect with someone else with fewer consequences for being open and honest: “*I called a help line a few times. eheadspace with chats*… *just talking to someone who will actually listen but not tell your parents. It’s weird talking to friends sometimes*” (Pt 1). While participants found online forums helpful, they also cautioned about their use: “*Definitely helpful*… *just need caution when putting people together with the same issues there is a level of support it gets to but then a level of enabling it can get to*” (Pt 7).

Participants reported that getting out (theme 4) into public places, and doing helpful activities such as exercise or going to helpful environments (i.e., friend’s house) was effective when there was an urge to self-harm. Being in a public environment, structured setting, or in the presence of others, meant they were removing themselves from a trigger and were not able to engage in self-harm. “*Was helpful to be places where there was structure and I couldn’t leave. in public I wouldn’t harm myself, because what would happen then? What would people do?*” (Pt 2), “*if I exercise I am in a much better head space*” (Pt 3), “*Because often is triggered by something that isn’t real etc*… *so need to figure out emotion and then back to the actual problem*… *so removing yourself gives you the opportunity to do this*.” (Pt 6).

Mimicking strategies (theme 5) were actions participants described that provided the same sensation as self-harm but without the consequences (e.g., flicking a rubber band around the wrist, holding an ice-cube in the hand). These were mostly seen as unhelpful; however, one participant highlighted their importance for use in public places: “*It doesn’t give you the same feeling but is ok when out*” (Pt 2). Mostly, participants described these mimicking strategies in negative ways: “*These strategies [rubber band and ice] are condescending*… *don’t really communicate how valid my distress is*” (Pt 4); “*Those things [flicking a rubber band] aren’t really the same in terms of they don’t really hurt*… *the blood is important*… *and getting the physical pain to mask the emotional pain is important*.” (Pt 2); “*I tried the elastic band for a while*… *but I got carried away and I did it all the time. But it actually made me self-harm in some ways*… *I kept doing it worse and worse*.” (Pt 6).

### Recommendations for Developing an Online Intervention for Young People at Risk of Self-Harm

An online intervention and particularly an app was described as a potentially useful tool that could be a place to store everything that might be helpful for managing the urge to self-harm. Participants described a range of potentially useful things that could be included in an app, such as pictures and videos that made them feel good, laugh, or express other emotions, online games useful for distraction, reminders of people who love and care for them, and links to other apps to support self-help strategies like mindfulness and meditation. Participants were enthusiastic about a function within a developed intervention that would allow them to share self-help strategies with others, stating that hearing about strategies from other young people with lived experience was preferred: “*It is support from people who actually know what it is like to go through stuff and who have tried stuff suggested and have had success, and also acknowledging that it might not work for everyone but it is worth a try*” (Pt 7). Connecting with people who have similar experiences was also described as potentially helpful, although participants were mindful of the risk: “*‘There is still hope’ journey stories. Having links to this sort of thing would be useful*… *to have a playlist of stuff exploring mental illness/health*… *but this might be triggering for some people*.” (Pt 5). Participants described the importance of having regular reminders to use these strategies, as well as a prompt for use if they became distressed.

Ways for overcoming barriers to social connection were also discussed as an important function of an intervention: “like *the idea of an option to get the app to send a message to nominated person to say you aren’t doing so well*… *so they can contact you and initiate the conversation*” (Pt 2), although participants were keen to maintain control over this type of function: “*mustn’t be automatic*… *needs a box that says looks like you’re having a bad day do you want this info passed to X*.” (Pt 1). Related to this, participants stated that a function that allowed them to send a prewritten message to a nominated support person when they felt distressed would be useful: “*When you are in that space it is too hard to process anything, so you need stuff that is already there and ready to go*.” (Pt 3). Participants noted the potential for social networking in terms of facilitating a supportive community to allow positive feedback on achievements and to share helpful strategies but acknowledged the potential for harm and suggested that moderation would be needed to ensure no triggering content was posted.

In addition, participants highlighted the need for customization, for example by applying particular restrictions on available content dependent on an individual’s own triggers, the ability to modify when push notifications were sent reminding them to use strategies, and accommodating people at different stages of recovery.

## Discussion

This study is among the first to explore the lived experience of young people who have engaged in self-harm and is unique in its examination of immediate triggers of the urge to self-harm as opposed to generic risk factors for self-harm. This research adds to the literature on self-help strategies that can be used “in the moment” to mitigate the urge to self-harm, by providing strategies that young people have found useful in overcoming emotional distress and feeling more socially connected.

Experiencing distressing emotions and a sense of isolation were key themes, which were considered primary triggers, because other triggers were associated with their induction (i.e., participants reported that exposure to self-harm, relationship difficulties, social comparison, and school/work difficulties lead to distressing emotions and a sense of isolation, which in turn triggered the urge to self-harm). That psychological distress is a trigger for self-harm is consistent with previous research (Landstedt and Gådin, 2011; Townsend et al., 2016), but this study highlights the wide range of distressing emotions reported by young people as triggering, and that these emotions arise from an equally wide range of situations.

A sense of isolation was reported to arise in a range of contexts but commonly young people described feeling isolated when they perceived themselves as different to others (e.g., in terms of sexual orientation, having a diagnosed disorder). This may go some way to explaining the mechanisms that lead to what we know are higher rates of self-harm in these populations (4). Unfavorably comparing oneself to perceived social norms, either in person or via social media, was reported to lead to a sense of not fitting in. In addition, the young people who reported feeling less supported by, or able to confide in, parents, teachers, or peers, reported that this could leave to an urge to self-harm, which is a finding consistent with previous research (Portzky et al., 2008; Swahn et al., 2012). Previous research has also found an association between parental criticism and self-harm (Yates et al., 2008), and in the current study, young people mentioned that feeling disconnected from or unsupported by family, experiencing relationships difficulties and conflict, was a trigger for self-harm. The reports from young people in the current study suggest that the resulting sense of isolation or distressing emotions act as mediator between relationship difficulties and conflict and self-harm. Thwarted belongingness (in our study “social isolation”) is a key component of Joiner (2005) interpersonal theory of suicide, the risk of which is elevated in those who engage in self-harm. Understanding how Joiners theory might apply to self-harm may be a useful direction for the field (3).

Being exposed to others self-harm was identified as a trigger for self-harm, which is consistent with the literature (McMahon et al., 2013; O’Connor et al., 2014), but the study highlighted significant nuances with regard to how this exposure might trigger a young person to engage in self-harm. Comparing the extent of injury could act as a trigger for young people who felt their injury was not severe enough in comparison; a sense of shame at a perceived sense of their own problems not being as bad as those they were reading about was reported as triggering, and empathizing with others’ distressing emotions and negative experiences was also considered triggering. Previous research has shown that graphic images lead to comparison and competitiveness regarding the severity of wounds, and that graphic images can lead to young people believing self-harm is an acceptable coping method (Lewis and Baker, 2011; Baker and Lewis, 2013).

A key theme from this study, perhaps not surprisingly given the findings about the very wide range of situations and emotions that can be triggering, was the idiosyncratic nature of the self-help strategies young people found helpful. Young people were adamant that what might be deemed helpful will vary according to the individual, their environment, the triggering situation, mood, and level of distress. This finding makes sense in the context of research showing that individuals engage in self-harm for a variety of reasons (Rodham et al., 2004; Claes and Vandereycken, 2007). Therefore, an important finding of this study is that strategies to support young people who self-harm must address these complexities. For example, participants highlighted that distraction strategies needed to be matched to the type and intensity of the distressing emotion experienced. Young people highlighted the need to ensure that a range of options is available to choose from and that those using the strategy would need to select what was appropriate to their experience and the particular situation they found themselves in.

Building connections with others was reported by young people in this study as an important strategy for overcoming a sense of isolation, which was identified as an important trigger. However, consistent with previous research (Doyle et al., 2015), many participants described finding communicating with others particularly challenging, especially when they were distressed. Participants wanted channels and templates to support them in making connections when they needed them most and provided a number of useful suggestions for a digital intervention to support social connectedness through messages to significant others and a moderated chat function. In terms of formal therapeutic interventions for young people at risk of self-harm, inclusion of the family and other supports is often emphasized (Fortune et al., 2008; Cox and Hetrick, 2017; Cottrell et al., 2018), and this study extends this to highlight the need for young people to maintain some autonomy and control in terms of with whom and how they share their distress with others. Relevant to building connection, young people also described “changing the environment” as an important strategy for managing urges to self-harm, and this often involved going outside or going out with friends to engage in activities. Changing the environment is a strategy that is particularly well supported by previous research as it not only facilitates engagement with others – reducing feelings of isolation – it also engages behavioral activation, a key component of cognitive behavioral therapy, which involves small achievable tasks that increase positive affect by creating a sense of mastery or change in focus (Hopko et al., 2003).

This study was unique in being able to evaluate young people’s experience of “mimicking strategies,” which are often recommended by clinicians. Participants in this study acknowledged that they can be useful as a discrete method to be used when in public. However, they also noted that mimicking strategies were inadequate in the face of severe distress, and can be perceived as potentially condescending and invalidating.

### Strengths and Limitations

The participants in this study were help-seeking individuals who had sought professional help through secondary and tertiary mental health services as well as through general practice and private psychological services. Given this, these findings may not generalize to young people who engage in self-harm but have not sought help. For example, participants’ responses to mimicking strategies in the current study may reflect the nature of the sample who were help-seeking and in remission for 3 months and that these participants may have learnt more effective emotional–regulation strategies to deal with urges to self-harm than individuals who are yet to seek any help. While participants were all help-seeking, we did not systematically collect information about mental health diagnoses. Specific diagnoses may result in consistent reactions to particular kinds of triggers, but this was not explicitly explored. That our participants represented an array of diagnoses and psychosocial issues may also in part contribute to the finding about the idiosyncratic nature of self-help strategies. Finally, given the retrospective nature of the study, participants may have forgotten or inaccurately recalled details of triggers and helpful strategies.

Strengths included the systematic literature review undertaken to guide the development of the interview schedule to ensure all questions were relevant, and the robust frameworks followed for qualitative analysis.

## Implications and Conclusion

Young people at risk of self-harm require strategies to support them in moments when they are experiencing distressing emotions and have an urge to self-harm. Not withstanding that young people may require longer term interventions to support their journey to recovery, the insights from this research about “in the moment” or short-term strategies for managing the urge to self-harm are important. The research has highlighted that these strategies need to help young people manage distressing emotions and support them to connect with others. An important aspect of such strategies is helping young people to become aware of their triggers, including the contexts and situations, but also the arising psychological distress that precedes their urge to self-harm. This, for example, might be done by the person who is supporting the young people person via techniques such as functional or “chain analysis” that supports an individual to uncover all the factors that led to the urge to self-harm. Young people in this study identified a large number of potentially helpful strategies to manage self-harm urges, while highlighting that different strategies will work for different people, in different settings and at different times. Therefore, in terms of developing strategies to help them with the “in the moment distress” it is important that young people become aware of what most helps them. Digital interventions appear to offer great potential to support young people who self-harm, especially if they provide opportunities for a young person to think about their own individual triggers, offer a wide range of self-help strategies that they can choose from to manage the urge to self-harm, are customizable to an individual’s own triggers and strategy responsiveness, are moderated by a third party for safety, limit content about methods or scars, and facilitate a range of functions for improving social connections. Through its unique findings relating to the mediating role of distressing emotions and social isolation in prompting the urge to self-harm, the potential inadequacies of mimicking strategies, and alternate activities young people engage in and find helpful when experiencing the urge to self-harm, this research highlights the importance of individual strategies that help a young person to use strategies that assist with emotion regulation and to feel more connected to others.

## Data Availability Statement

The raw data supporting the conclusions of this article will be made available by the authors, without undue reservation, to any qualified researcher.

## Ethics Statement

This study was carried out in accordance with the recommendations of the National Statement on Ethical Conduct in Human Research (NHMRC, ARC, UA, 2007) (National Statement) with written informed consent from all subjects. All subjects gave written informed consent in accordance with the Declaration of Helsinki. The protocol was approved by the Melbourne Health Human Research Ethics Committee (HREC/15/MH/340).

## Author Contributions

SH and JR conceived on this project and developed the methodology. KA and AS undertook the research as part of the requirements of the Honours in Psychology (La Trobe University), and along with SH interviewed all the young people, transcribed the interviews, and took a lead in undertaking the analysis with input from all the authors. SH drafted the manuscript with substantial input from other authors who all approved the manuscript for final submission.

## Conflict of Interest

The authors declare that the research was conducted in the absence of any commercial or financial relationships that could be construed as a potential conflict of interest.
