# Supplementary material for: Understanding the Needs of Young People Who Engage in Self-Harm: A Qualitative Investigation
Source: Front Psychol. 2020 Jan 10;10:2916. doi: 10.3389/fpsyg.2019.02916 (PMC6968776; doi:10.3389/fpsyg.2019.02916)
Supplement: Supplementary file 1 [file Data_Sheet_1.docx]

Supplementary Material

**Findings from systematic review:**

Five questions (as well as associated prompts for use if a participant did not mention specific concepts in their responses), relevant to triggers for self-harm were derived from the literature synthesis. The synthesized literature highlighted relationship difficulties, distressing emotions, exposure to self-harm, and school/work difficulties as key factors and showed some inconsistency in how alcohol and drug use was relevant to being a triggers for self-harm. Questions were asked about potential online triggers of self-harm and were designed to identify what would be harmful in this environment.

Five questions, with associated prompts, relevant to potentially helpful strategies for managing the urge to self-harm were derived from the literature synthesis. The academic literature was largely focused on clinical interventions, such as specific psychological therapies, with little discussion of the effectiveness of specific techniques (particularly to manage the urge to self-harm) of these interventions. The components of these clinical interventions were broadly described as distress tolerance skills, emotion regulation skills, communication skills, strengthening relationships and involving families and supports. The online literature reported over 70 specific techniques that were grouped according to four functions: soothing emotions, distraction, connecting with others, mimicking self-harm. The focus of the five questions derived from the synthesized literature was to understand which of all the strategies and therapeutic interventions described, young people actually used and found helpful, how connection with family, friends and others could most usefully be achieved to assist the young person when they were experiencing the urge to self-harm, and how to present the many possible helpful strategies to young people in a way that facilitated engagement. The interview concluded with questions about recommendations for the development of an online intervention to support young people who self-harm, including how best to translate self-help strategies into digital interventions, and how these might be presented to young people at risk of self-harm.

On the basis of these findings the following semi-structured interview guide was developed.

**Semi-structured Interview guide**

**Triggers for self-harm**

1. **One of the themes to emerge from the literature was that experiencing difficulties with school and work life were problematic for some young people and was found to act as a trigger for self-harm. Did you experience something similar to this?**
2. **Exam stress**
3. **Employment issues**
4. **Similarly conflict with family and partners was a common trigger for self-harm in young people. Were there any specific situations, or feelings associated with that conflict with your family or partners that was directly related to feeling the need to engage in self-harm?**
5. **Based on my literature results I found that the most common trigger for self-harm was experiencing difficult emotions. Were there any specific emotions you felt prior to self-harm urges?**
6. **Loneliness and isolation**
7. **One of the major risk factors for self-harm as reported particularly by the academic literature was being exposed to the self-harm behaviours of family and friends. Do you know others in your family or friendship groups that have self-harmed?**
8. **Did knowing or hearing about or witnessing their self-harm behaviour result in you feeling the need to self-harm?**
9. **Were you exposed to self-harm through any other means that you found triggering?**
10. **Internet (pro-self-harm websites), movies, magazines**
11. **A proposed risk factor for self-harm is the use of alcohol or drugs. I just wanted to clarify how you would expect to find or perhaps did find the use of alcohol or drugs to be triggering? Or possibly make you more vulnerable to self-harm?**
12. **During the use of alcohol and/or drugs; after the use- coming down from high or hungover?**
13. **Were there any specific emotions involved? (regret, shame, disgust)**
14. **In your opinion/experience what kind of content would you imagine being triggering in an online environment?**
15. **Images, details about methods of self-harm, blood flow**
16. **Differences between different platforms/websites- most/least helpful**
17. **What influences whether previously helpful content becomes triggering in specific circumstances (emotions/moods)?**

**Helpful strategies to mitigate the urge to self-harm**

1. **What strategies have you found helpful in the moment to reduce the need to engage self-harm or effectively replaced to role of self-harm for you?**
2. **Writing in a journal, ice cube, rubber band, shower, music, exercise, calling friend (unrelated), strong taste, red pen, punching pillow. Activity: Post it notes**
3. **What function it served for you (distract, soothe, express)**
4. **What has been your experience or do you have any thoughts on the possible helpful role of family and friends when there is an urge to self-harm?**
5. **would you use family and friends**
6. **what would be an acceptable way to engage their help**
7. **Is this something that can be used online**
8. **Literature has highlighted the importance of having support from others and a sense of connection with other people. What ways have you found to be effective to help you feel connected with and supported by others?**
9. **specifically are there online ways to achieve this**
10. **chat rooms, blogs, social media**
11. **Time when you have sought support and connection online and this has not helped? (Judgement, fix it mentality)**
12. **What would be an acceptable way to communicate**
13. **There are over 70 different strategies that were reported to be useful in the online information. In your experience what would be the most effective way to communicate these strategies? Either broad concept or very specific actions? For example exercise or specifically vigorous work out, yoga, walking etc. and what form i.e. activity diary, safety, monitoring**
14. **How would you use the information; Safety planning, mood/activity diaries, playlists**
15. **A strategy commonly explored in therapy is using mindfulness techniques to reduce and manage overwhelming emotions? Have you experienced this to helpful and what other ways you have been able to cope with overwhelming emotions instead of using self-harm?**
16. **positive activities, opposite actions, meditation, breathing**
17. **Do you have any activities you can share that you do online and are positive for you in general?**
